# The Progression of Chronic Myeloid Leukemia to Myeloid Sarcoma: A Systematic Review

**DOI:** 10.7759/cureus.21077

**Published:** 2022-01-10

**Authors:** Hadia Arzoun, Mirra Srinivasan, Santhosh Raja Thangaraj, Siji S Thomas, Lubna Mohammed

**Affiliations:** 1 Internal Medicine, California Institute of Behavioral Neurosciences & Psychology, Fairfield, USA

**Keywords:** blastic crisis, hematology, chronic, disease progression, myeloid sarcoma, chronic myeloid leukemia

## Abstract

Chronic myeloid leukemia (CML) is a slow-growing type of cancer that originates in the blood-forming cells of the bone marrow and is caused by a chromosomal mutation that is thought to occur spontaneously. CML could potentially lead to the development of myeloid sarcoma (MS), which is a rare neoplasm composed of immature myeloid cells that could evolve into a tumor mass at any anatomical site other than the bone marrow. MS can develop spontaneously or as a result of another form of myeloid neoplasm. Most instances of CML precede blast phase (BP) within two to three years after the first diagnosis of CML chronic phase (CP) at the age of pre-tyrosine kinase inhibitor (TKI) treatment. MS developing in CML patients during the era of TKI treatment is infrequently mentioned in the literature, primarily in single-case studies. As a result, the prognostic influence of MS in CML patients has not been well investigated. In the age of TKI treatment, it is uncertain whether MS and medullary BP have comparable clinical and prognostic relevance. The precise diagnosis of MS is critical for effective treatment, which is frequently delayed due to a high risk of misdiagnosis.

This review focuses on the relationship between the development of MS from CML, and it culminates with recommendations for future hematology practice. A literature search was conducted in multiple databases, and the studies were appraised based on the inclusion and exclusion criteria.

Finally, studies to date have shown that the existence of CML and its possible progression to MS in individuals map out the numerous implications this disease has in hematology practice. Though occurrences are uncommon in general, the prognosis for patients is bleak, necessitating the exploration and implementation of diagnostic and therapy advancements. Because there is limited evidence in the literature on its existence in the medullary chronic phase and outcomes in the era of TKI, it must be carefully investigated because it might be the first symptom of progressive illness prior to hematological progression.

## Introduction and background

Diseases of the blood can have significant acute and chronic impacts on the patient's quality of life. The field of hematology-oncology has experienced vast levels of growth in recent decades and has enhanced the treatment modalities available for cancerous blood conditions, such as leukemia [1]. One common form of leukemia impacting adults in contemporary society includes the onset of acute myeloid leukemia (AML), which accounts for an estimated 80% of cases of leukemia [2]. In many cases, the presence of a chronic form of myeloid leukemia, known as chronic myeloid leukemia (CML), can ensue, impacting the functioning of white blood cells in afflicted patients. The presence of CML can impact the central nervous system (CNS) or manifest in the development of myeloid sarcoma (MS) [3].

Myeloid sarcoma, also known as granulocytic sarcoma or chloroma, is a rare buildup of malignant myeloid progenitor cells in an extramedullary location that affects the typical architecture of the affected tissue [4]. The word "chloroma" comes from the Greek word "chloros" meaning "the color green" [5]. It shows the tumor's greenish tint caused by the presence and oxidation of the enzyme myeloperoxidase [6]. These tumors can develop independently or in conjunction with other myeloid illnesses such as AML or CML, myeloproliferative or myelodysplastic syndromes [6]. The presence of myeloid sarcoma results from a developing tumor created by hematopoietic neoplasm [7]. It can originate anywhere, resulting in a wide range of clinical manifestations [8]. Bone, skin, and lymph nodes are the most prevalent areas of involvement. Rare occurrences, however, have been observed in the gastrointestinal tract, genitourinary tract, or breast [4]. The diagnosis is complex and requires a high index of suspicion, as well as radiography, histology, immunophenotyping, and molecular analysis, which are also required for risk stratification and treatment planning [7].

Though a myeloid sarcoma can develop independently, it is often attributed to an underlying form of leukemia, such as CML [9]. In order to investigate the disease and its progression in greater depth, research was conducted on the existing knowledge around pathology, presentation, diagnosis, and treatment of both myeloid leukemia and myeloid sarcoma [7]. The following review intends to connect this data with the progression from chronic myeloid leukemia to myeloid sarcoma and conclude with the implications for the future hematology practice.

Methodology and results

This systematic review follows the Preferred Reporting Items for Systematic Reviews and Meta-Analysis (PRISMA) guidelines and principles [10].

Information Sources and Search Strategy

A literature search was done in PubMed, PubMed Central, and Google Scholar. A total of 1,570 articles were found using the keywords that are listed below. After removing all the duplicates, the reports were screened according to the inclusion and exclusion criteria set by the authors (which is described below), and all irrelevant reports were eliminated. The study team used appropriate quality assessment tools to evaluate if the studies met the inclusion criteria during the selection process. Data selection and extraction were carried out independently by two researchers between October 25, 2021, and November 6, 2021, from the three databases, indicated above. In cases of discrepancies, both researchers addressed the study designs, inclusion and exclusion criteria, intervention used, and outcome measurement in order to achieve an agreement. In ambiguous cases, a third reviewer was engaged to resolve conflicts and reach a consensus. Upon completion of this comprehensive process, eight reports were finally included for this review.

Inclusion and Exclusion Criteria

This study's inclusion criteria comprised full-text English language reports published in the previous ten years (2011-2021), with a global search of findings encompassing observational studies, case reports, and experimental research. Non-English, non-full-text literature, research conducted prior to 2011, and animal experiments were excluded. This study included the population, intervention, comparison, outcome, and study criteria (PICOS), with the population group consisting of individuals (female and male) from a multi-cultural background.

Keywords: MeSH keywords in PubMed are as follows: Chronic myeloid leukemia OR chronic myelocytic leukemia OR ("Leukemia, Myelogenous, Chronic, BCR-ABL Positive/analysis"[Mesh] OR "Leukemia, Myelogenous, Chronic, BCR-ABL Positive/complications"[Mesh] OR "Leukemia, Myelogenous, Chronic, BCR-ABL Positive/drug therapy"[Mesh] OR "Leukemia, Myelogenous, Chronic, BCR-ABL Positive/mortality"[Mesh] OR "Leukemia, Myelogenous, Chronic, BCR-ABL Positive/pathology"[Mesh] OR "Leukemia, Myelogenous, Chronic, BCR-ABL Positive/therapy"[Mesh] ) AND myeloid sarcoma OR granulocytic sarcoma OR ("Sarcoma, Myeloid/analysis"[Mesh] OR "Sarcoma, Myeloid/complications"[Mesh] OR "Sarcoma, Myeloid/diagnosis"[Mesh] OR "Sarcoma, Myeloid/diagnostic imaging"[Mesh] OR "Sarcoma, Myeloid/etiology"[Mesh] OR "Sarcoma, Myeloid/pathology"[Mesh] OR "Sarcoma, Myeloid/therapy"[Mesh]).

Keywords in other databases are as follows: Chronic myeloid leukemia; myeloid sarcoma; disease progression; chronic; hematology; blastic crisis.

Results

**Figure 1:**
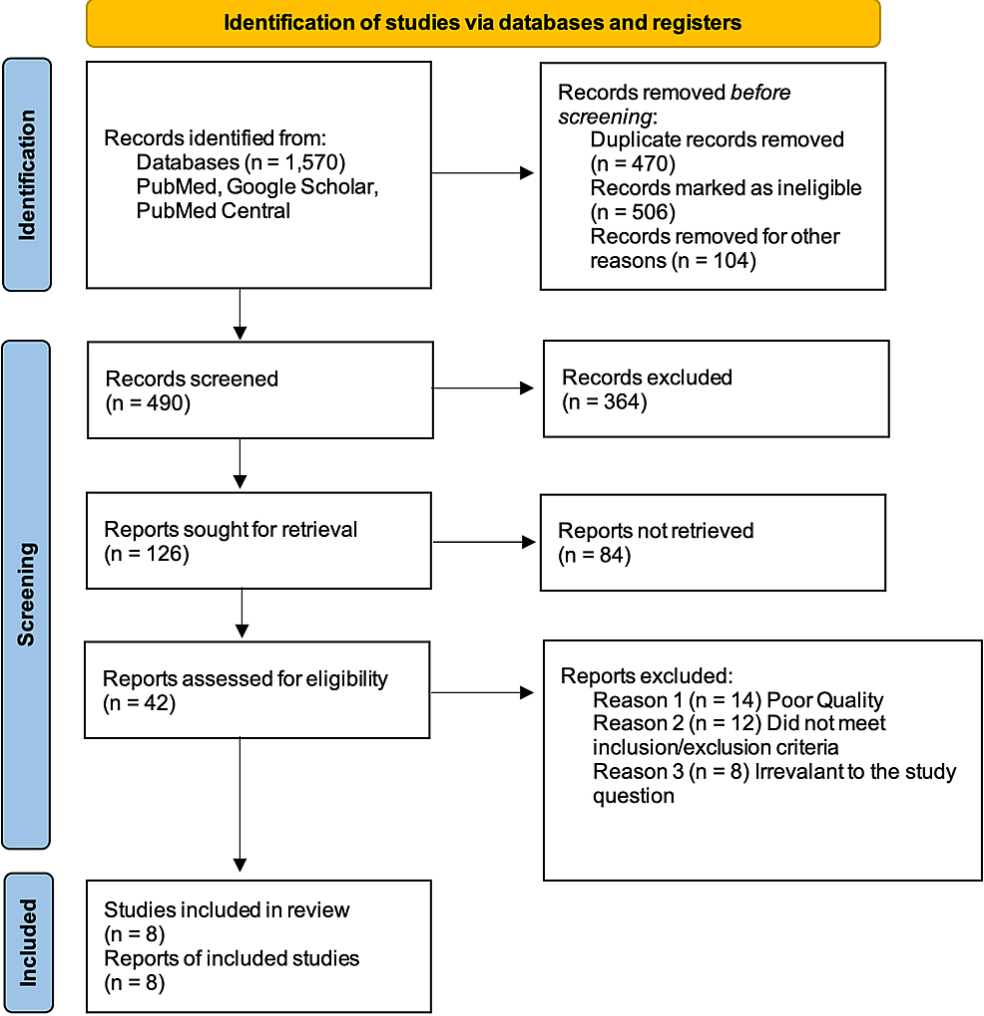
PRISMA 2020 flow diagram for new systematic reviews includes searches of databases and registers only. PRISMA: preferred reporting items for systematic reviews and meta-analysis.

As aforementioned, the search strategy used in this study included three different databases, yielding 1,570 articles, of which 470 were duplicates and were removed using Microsoft Excel, 506 articles were removed due to ineligible records where the title/abstract was not relevant to the research question, and 104 records were removed for other reasons, like excluded study designs and irrelevant research topics. A total of 490 records were reviewed, with 364 being discarded due to relevancy and inclusion/exclusion criteria. Eighty-four reports could not be retrieved, and the final screening reduced the number of reports to 42, which were evaluated for quality and eligibility. The evaluation comprised eight studies after a thorough reading. The study did not use any automation tools. Figure 1 depicts the search process used for this review in the form of a PRISMA flow diagram [10].

Quality Assessment of the Studies

Table 1 below displays the kinds of studies assessed and the appropriate scores assigned to each study based on the quality appraisal tools [11,12].

**Table 1:** Assessment of included studies along with overall appraisal score. JBI: Joanna Briggs Institute.

Type of study	Number of studies	Quality appraisal tool	Overall appraisal/scores
Case report [11]	3	JBI critical appraisal checklist for case reports	Include (≥7)
Retrospective cohort [12]	5	Newcastle-Ottawa	Include (≥10)

Focal Points of the Selected Studies

The final studies included in this report are summarized in Table 2 below.

**Table 2:** Focal points of the included studies. MS: myeloid sarcoma; MyBP: medullary myeloid blast phase; CML: chronic myeloid leukemia; TKI: tyrosine kinase inhibitor; AML: acute myeloid leukemia; CML-CP: chronic myeloid leukemia-chronic phase; CML-BP: chronic myeloid leukemia-blast phase; HSCT: hemopoietic stem cell therapy.

Author/year	Type of study	Sample size	Findings	Conclusion
Chen et al. (2016) [13]	Retrospective Cohort	307	Although MS and medullary MyBP that develop late in the course of treatment have a similarly poor prognosis, patients with MS without MyBP at initial diagnosis had a significantly better outcome and contributed to the overall better prognosis of patients with MS than those with CML in medullary MyBP.	In the period of TKI treatment, MS has become less prevalent in people with CML.
Kawamoto et al. (2016) [14]	Retrospective Cohort	131	MS patients tended to lack myeloid markers (myeloperoxidase was present in 63.2%, CD68 in 51.3%, CD13 in 48.7%, and CD33 in 48.7% of patients) and express T-cell markers such as CD3 in 20.7% and CD5 in 34.2%. In immunohistochemistry, all T-cell marker-positive MS subjects were negative for T-cell receptors.	There is no statistically significant difference in prognosis between de novo and concurrent AML (P = 0.288) and no statistically significant changes in prognosis between T-cell marker-positive and T-cell marker-negative MS patients.
Dasappa et al. (2017) [15]	Retrospective Cohort	615	Eight CML-CP patients developed MS. With larger doses of imatinib/nilotinib, the median overall survival was 14 months, with the highest survival of 36 months in a case of nilotinib. In contrast, four patients advanced to MyBP for a median period of nine months and died.	MS had a better prognosis in medullary CML-CP than in medullary CML-BP.
Vasconcelos et al. (2017) [16]	Case Report	1	The identification of myeloid sarcoma lesions allowed the dermatologist to eventually exercise the initial perception of the lymphoproliferative disease and promote a multidisciplinary approach.	A 42-year-old female was diagnosed with chronic myeloid leukemia, which was confirmed by a skin biopsy.
Zhou et al. (2019) [17]	Retrospective Cohort	33	MS with non-favorable cytogenetics in pediatric patients had a significantly lower overall survival than patients with AML with non-favorable cytogenetics and no extramedullary involvement (P < 0.001).	For pediatric MS patients, non-favorable cytogenetics, documented as abnormalities other than t (8;21), inv (16)/t (16;16), or t (15;17), may be a poor prognostic indicator.
Palejwala et al. (2019) [9]	Case Report/Review Article	1	Complete excision of an intraparenchymal myeloid sarcoma was done in a female with a history of CML on imatinib (non-compliant) with a relapse to blast crisis.	A 50-year-old female’s acute neurological symptoms were completely resolved after surgery, with no postoperative complications.
Lee et al. (2020) [18]	Case Report	1	At the time of the CML diagnosis, MS was asymptomatic. However, shortly after starting imatinib, a hematoma formed in the tumor, causing muscular strain and soreness. MS was diagnosed as a result of the pain produced by the enlarged thigh circumference. It arose during the chronic phase of CML and comprised all myeloid lineages.	A 16-year-old male had coexisting MS and CML-CP.
Frietsch et al. (2021) [19]	Retrospective Cohort	307	The median onset of MS was 425 days post-HSCT, and the median survival was 234 days.	MS following HSCT is associated with a poor prognosis, as multimodal therapeutic approaches such as rigorous chemotherapy and additional HSCT are frequently ineffective.

## Review

This section of the review aims to enumerate what myeloid leukemia is, including the pathogenesis, clinical presentation, diagnosis, and treatment, while also discussing myeloid sarcoma and its pathology with clinical presentation, diagnosis, and treatment. Finally, an in-depth analysis of the progression of myeloid leukemia to myeloid sarcoma is also discussed, as well as the limitations of this study.

An overview on myeloid leukemia

Myeloid leukemia comes in two major forms, including acute and chronic. In cases of AML, patients afflicted often have an underlying hematological disorder, and the onset of the disease is rapid. AML is the most common form of leukemia, comprising approximately 80% of all cases [2]. Alternatively, CML cases are rarer in their occurrence and comprise an estimated 15% of adult leukemia cases [20]. CML is typically present in approximately 10 to 15 cases per million annually in the global population and demonstrates no ethnic distinction among patients diagnosed. In European nations, the age of onset is typically noted between the ages of 60 and 65. However, in many nations with younger populations, the diagnosis of CML occurs in younger age ranges [21]. Alternatively, in the United States, CML demonstrates an incidence of approximately one to two new cases per 100,000 adults each year [20]. Furthermore, unlike AML, CML progresses more slowly over an extended period before the patient enters the accelerated and blast phases [21]. Important details regarding the pathology, presentation, diagnosis, and treatment are essential to explore prior to the discussion of its progression to and development of myeloid sarcoma.

Pathology

Normal hematopoiesis is defined by the existence of hematopoietic stem cells capable of regulated self-renewal and multipotency, resulting in balanced hematopoiesis between myeloid and lymphoid lineages [22]. The pathology of CML cases is attributed to myeloproliferative neoplasm, a rare blood cancer characterized by rapidly growing and unregulated myeloid cells [2,20]. When hematopoietic stem cells (HSCs) acquire the transformative mutation known as the breakpoint cluster region gene-Abelson proto-oncogene (BCR-ABL) (Vuelta) due to genetic change occurring between chromosomes nine and 22 [23], the cells gain uncontrolled self-renewal ability and develop into leukemic stem cells (LSCs), which results in CML [22].

**Figure 2:**
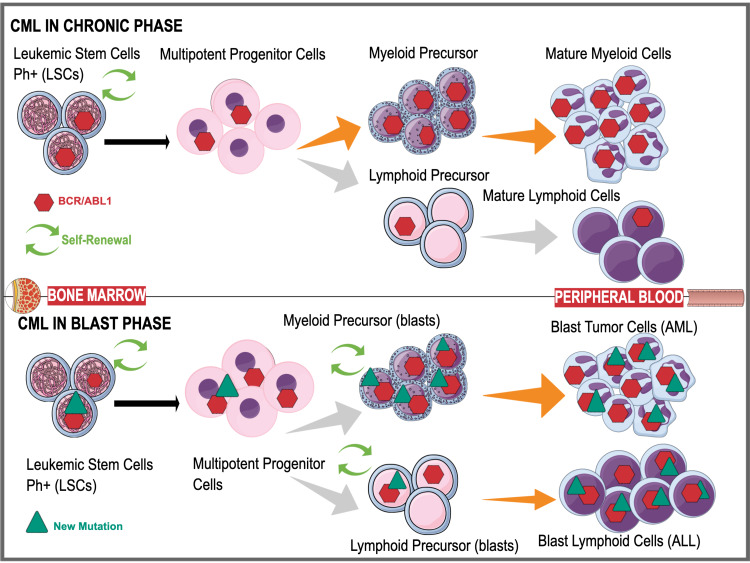
Chronic myeloid leukemia in chronic and blastic phase. CML: chronic myeloid leukemia; BCR-ABL: breakpoint cluster region gene-Abelson proto-oncogene; AML: acute myeloid leukemia; ALL: acute lymphoblastic leukemia. Mind the Graph Platform was used to create this figure.

The genetic changes between the two chromosomes involve a shortening of chromosome 22 caused by the formation of the BCR, which impedes its essential relationship with chromosome nine, resulting in a disease of hemopoietic stem cells [20,21]. It is to be noted that chromosome nine is also impeded by the fusion of the ABL1 gene [20]. Though scientists hold a strong understanding of molecular pathogenesis, the causation of the genetic change between the two chromosomes remains unknown [21]. However, the pathology described has been beneficial in identifying treatment modalities and the future progression of CML in afflicted patients. Additional genetic alterations can occur after establishing BCR-ABL in CML chronic phase (CP), and the illness eventually develops into the catastrophic CML blastic phase known as a blastic crisis (BC) [24]. A maturation stop in the myeloid or lymphoid lineage characterizes a blast crisis. New genetic and epigenetic abnormalities emerge in leukemic stem cells (LSCs), and blast cells migrate from the bone marrow to the peripheral circulation. Figure 2 below depicts the clinical phases of chronic myeloid leukemia [22].

Presentation and diagnosis

CML has a distinct and notable presentation. Typically, patients experience the onset of chronic anemia, malaise, weight loss, fatigue, pain or fullness in the upper left quadrant, and bleeding issues in more rare cases [20]. The progression of the disease, as mentioned above, often results in a progression from the chronic stage to the accelerated stage and the final blast stage, which results in uncontrolled levels of white cells in the body and the continuation of worsening symptoms. The diagnostic process involves documenting CML symptoms that can then be verified with the aid of blood count characteristics performed through lab draws [20,21]. Genetic testing often confirms the diagnosis through the identification of the changed chromosomes and BCR-ABL1 transcripts. These are typically obtained through bone marrow cells or peripheral blood [21].

Treatment

CML has been treated in various ways over the last several decades. One treatment route involved sole observation of the patient and their symptomatic presentation and progression [25]. Alternative treatment routes have included the provision of chemotherapy with medication administration of either hydroxyurea or busulfan. In other cases, stem cell transplants have been conducted, as well as the provision of tyrosine-kinase inhibitors (TKIs) and interferon-alpha (INFx) administration [25]. In recent years, combined therapy approaches have often been employed. This includes TKI and interferon combinations and TKI and chemotherapy combinations to enhance the effectiveness of treatment approaches. Additionally, the provision of immune modulation has also been incorporated into treatment options with a strategy to provide vaccinations to induce the desired response to leukemia activity [26]. Despite the employment of each, there is no cure for CML to date.

An overview on myeloid sarcoma

Myeloid sarcoma (MS) is a rare malignant tumor growth that occurs in approximately two cases in every million adults. The onset of MS has been noted to impact males at a higher rate than females and is often associated with the development and progression of AML. Patients who have developed CML also have an increased risk of developing MS, impacting approximately 9% of patients diagnosed with AML or CML [7]. Due to its relationship with CML, it is essential to briefly explore the pathology, presentation, diagnosis, and treatment of CML prior to discussing the process involved in the progression of CML to MS.

Pathology

The typical pathology of MS is attributed to the development of AML, myelodysplastic syndrome, or myeloproliferative neoplasm, including CML. However, in many cases, MS may be noted as a clinical presentation of AML and be detected prior to its development [27]. The physical presentation of MS often revolves around detecting the tumorous growth, green in color, which is considered granulocytic and extramedullary in nature [27,28]. The green coloration of the tumor is attributed to the presence of oxidation taking place within the granules of the tumor. Further, the extramedullary tumor is believed to develop due to impacts on the non-hematopoietic tissues by groups of leukemic myeloblasts [29]. Therefore, diagnosis upon presentation may be an important factor for the diagnosis of other hematological conditions, such as CML.

Presentation, diagnosis, and treatment

The diagnosis of MS is not always an easy task due to its capacity to grow within soft tissue portions of the body [7]. The presentation characteristics, therefore, are dependent upon the location and the impact on the organ affected [27]. For instance, one case study noted the development of MS within the mandibular gingiva region of the body and was noted after a molar extraction failed to heal appropriately [30]. Alternatively, a rare pediatric case received an MS diagnosis after swelling was experienced in the temporal lobe of a two-year-old patient [31]. Had the extraction not faced challenges in healing and swelling in the temporal lobe, the identification of the tumor and treatment may have been delayed in both cases. The presentation and diagnosis of MS are often noted in the soft tissue regions of the head and lymph nodes [32]. However, other sites noted as common regions for MS development include connective tissue regions, skin and breast tissues, and the digestive system [33]. To properly diagnose MS, symptomatic presentation is documented and followed up by tissue biopsy and radiological investigations [7]. Furthermore, treatment often involves chemotherapy approaches, bone marrow transplants, surgical excision, and targeted therapies tailored to the region affected by the tumor [7,34]. 

Progression of myeloid leukemia to myeloid sarcoma

**Figure 3:**
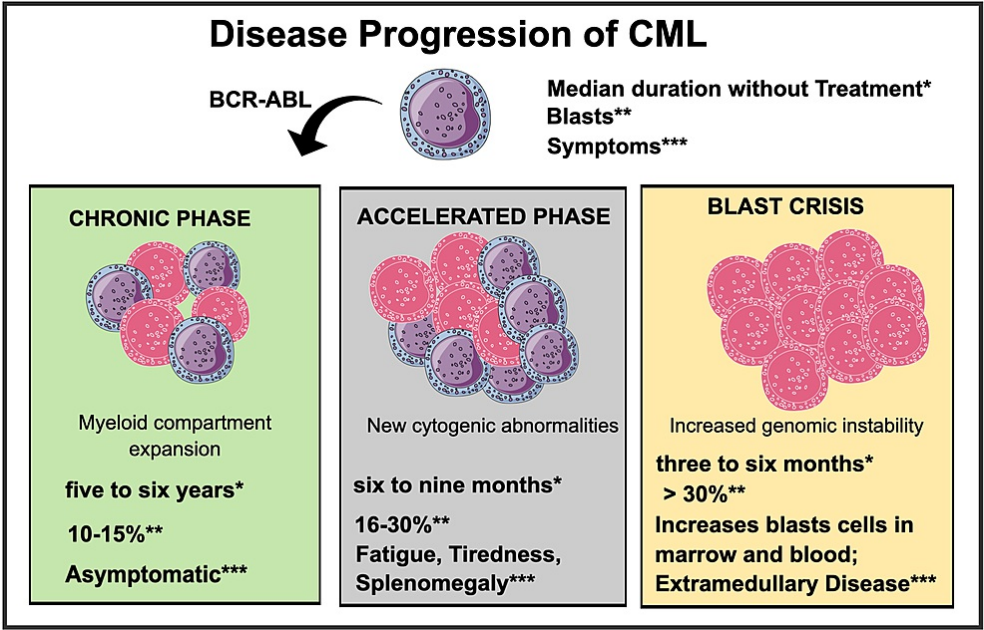
Disease progression of chronic myeloid leukemia. Mind the Graph Platform was used to create this figure. CML: Chronic myeloid leukemia, BCR-ABL: breakpoint cluster region gene-Abelson proto-oncogene. *Median duration without treatment; **percentage of blasts; ***symptoms.

In cases of CML, the presence of MS may precede a diagnosis of CML, though the progression of CML is typically the causal factor in the development and progression of MS [15,18]. The majority of CML patients are identified in the chronic phase, which will advance to the accelerated phase (AP) and, if left untreated, lead to a blast crisis. The quantity of immature cells (blasts) detected in the bone marrow characterizes each phase. BCR-ABL expression stimulates many signaling pathways in the myeloid compartment, resulting in enhanced proliferation and reduced apoptosis [24]. Secondary genetic and molecular defects cause an accumulation of mutations and genomic instability, culminating in a blast crisis and a dismal prognosis for the patient. Figure 3 below illustrates the evolution of the CML disease [24].

Once the condition has progressed to the blast stage, the risk of the development of MS in patients increases to between 7% and 17% [13]. This is due to the pathology of MS and its tumor formation on blasts developed during the BP stage of CML [19]. However, patients often first progress from the chronic stage of the disease to the accelerated phase (AP), which is almost exclusively attributed to the onset of CML, though they can present as sole cases on rare occasions. The survival rate of patients diagnosed with CML is estimated to be around five years on average. Alternatively, once the disease has progressed to the AP stage, the prognosis is reduced to approximately six to nine months. The progression to the BP stage reduces life expectancy by three to six months on average [15]. The identification of MS may therefore indicate disease progression into the final stages of the disease unless remission is achieved through treatment.

The presence of MS can also manifest as a relapse condition in patients with established cases of CML [19]. For instance, some case studies note that treatment success resulted in CML remission with relapses occurring and blast crisis. During this process, the patient experienced a hemorrhagic infarct that resulted in the cystic development of a granulocytic sarcoma, or MS [9]. However, the progression of MS from CML can differ depending on whether the disease progression is medullary or restricted to the bone marrow [13]. Therefore, the presence of MS can indicate a relapse in the treatment and remission process, with progression occurring during the remission period.

In some cases, the presentation of MS can be noted as a presenting symptom of CML [15]. The CNS can often provide sanctuary to CML due to the lack of effectiveness present with some treatments once they contact the cerebrospinal fluid (CSF). The result of the CNS protection of CML is the manifestation of MS, though the presence of MS is not likely to impact the CNS and is more often attributed to the underlying presence of leukemia [9]. Upon detection of an MS tumor, the patient is often in the blast crisis stage of CML, though it may also be present during the chronic stage and provide the initial diagnosis and detection of CML [18]. In cases where the patient is in a chronic stage of MS development, hematological progression of CML has not yet taken place. However, cases such as these are commonly noted in medullary forms of CML, making them a distinct component of disease progression overall [15]. Further research is required to assess a more obvious route of progression in MS cases involving CML in the future.

A 42-year-old African-descent woman was studied in Brazil for a rare case of cutaneous myeloid sarcoma associated with chronic myeloid leukemia [16]. The identification of myeloid sarcoma lesions led to the diagnosis of leukemia in this individual, which was later confirmed by the demonstration of BCR/ABL rearrangement in peripheral blood through the polymerase chain reaction (PCR) [16]. Further immunohistochemical analysis revealed that the tumor was positive for CD43, lysozyme, and myeloperoxidase but negative for CD3, CD20, and CD30, supporting the diagnosis of myeloid sarcoma. Thus, after treating her leukemia with imatinib mesylate, her cutaneous lesions improved, and her white blood cell count normalized [16]. Zhou et al. research followed 33 MS patients from one month to 18 years of age for 32 years (1984-2016). Not only did they find concurrent or recurrent AML, but they also found one patient with atypical CML, with the skin being the most prevalent region of MS involvement [17].

Limitations

The limitations of this study are that most reports about this disease are retrospective and involve a small number of patients, thus not enabling the practitioners to rely on all studies due to the rarity of the illness. More prospective studies and randomized controlled trials with a larger patient population could yield a better understanding of the association between chronic myeloid leukemia and myeloid sarcoma.

## Conclusions

The presence of CML and its potential progression to MS in afflicted patients carries multiple implications for hematology practice. One implication includes the need to conduct further research to investigate the development and progression of CML to MS and assess the most effective treatment routes to reduce its occurrence. This is particularly true for MS cases due to their ability to manifest in soft tissue locations where they are not easily identifiable. Further, greater levels of observation must occur with patients in remission to reduce the risk of MS development and progression during this vulnerable time.
